# Dietary Patterns of Greek Adults and Their Associations with Serum Vitamin D Levels and Heel Quantitative Ultrasound Parameters for Bone Health

**DOI:** 10.3390/nu12010123

**Published:** 2020-01-01

**Authors:** Effimia Grigoriou, George Trovas, Nikolaos Papaioannou, Ismene Dontas, Konstantinos Makris, Konstantinos Apostolou-Karampelis, George Dedoussis

**Affiliations:** 1 Department of Nutrition and Dietetics, School of Health Science and Education, Harokopio University, El. Venizelou 70, 17671 Athens, Greece; efi_grigoriou@hotmail.com (E.G.); apostolou@bio.demokritos.gr (K.A.-K.); 2 Laboratory for Research of the Musculoskeletal System “Th. Garofalidis”, Medical School, National & Kapodistrian University of Athens, 10 Athinas Street, KAT General Hospital, 14561 Athens, Greece; trovas1@otenet.gr (G.T.); npapaioan@med.uoa.gr (N.P.); idontas@med.uoa.gr (I.D.); 3 Department of Biochemistry, KAT General Hospital, 14561 Athens, Greece; kostas.makris.km@gmail.com

**Keywords:** dietary patterns, Greece, 25(OH)D, QUS parameters, lifestyle

## Abstract

The aim of this study is to investigate the dietary patterns which indicate the nutritional habits of Greek adults and their effects on serum 25(OH)D levels and quantitative ultrasound (QUS) parameters for bone health. This study is part of OSTEOS, an observational cross-sectional study. In total, 741 adults from rural and urban areas throughout Greece were recruited. A validated food frequency questionnaire (FFQ) was used for assessment of the population’s dietary habits. Serum 25(OH)D was measured by enzyme immunoassay; QUS parameters were assessed with an Achilles device. Principal component analysis (PCA) was carried out for dietary pattern determination, and univariate analysis of variance was used for the assessment of 25(OH)D, broadband ultrasound attenuation (BUA), speed of sound (SOS), and stiffness index (SI) determinants. Six dietary patterns explain 52.2% of the variability of Greek adults’ nutritional habits. The ‘vegetables–fruit’ dietary pattern explains the biggest rate of variability. Determinants of serum 25(OH)D are body mass index (BMI), elderly status, summer sun exposure, organized physical activity, a ‘healthy’ pattern in winter months, and adherence to a ‘sweet’ pattern. Determinants of QUS parameters are age, BMI, sedentary time, organized physical activity participation, and adherence to a ‘healthy’ pattern.

## 1. Introduction

The role of nutrition in health and its effect on several phenotypes is widely known. Nutrient consumption and dietary habits affect bone health during the life span, both in peak bone mass achievement and its preservation during adulthood. Nutrients may have a beneficial action on the skeleton or aggravate the bone health [1]. The key role of several nutrients and food items like calcium, vitamin D, vitamin K, vitamin A, protein, polyunsaturated lipids, phosphorus, potassium, magnesium, caffeine, alcohol, and phytoestrogen, as well as dairy products, fruits, and vegetables in bone health has been thoroughly studied [2,3,4,5,6,7]. However humans consume a complex combination of nutrients that have a cumulative and interactive effect in their meals, related to several phenotypes or health outcomes. The need of a more holistic approach of nutrition led to the investigation of statistical methods from which overall dietary patterns can be derived, in which multiple related dietary characteristics are considered as a single exposure for a specific population. There are two main categories of dietary pattern approaches: (1) the data-driven or a posteriori dietary pattern approach that includes principal component analysis (PCA), and (2) the *a priori* dietary pattern approach, where dietary indexes used are based on existing nutritional knowledge [8,9].

Vitamin D deficiency is also an important risk factor of bone fragility. The main physiological effect of vitamin D is to increase intestinal calcium absorption, maintaining serum calcium in order to maximize metabolic functions. The consequences of vitamin D deficiency in adults’ bone health are osteomalacia, acceleration of bone loss, muscle weakness, instability, and therefore increased risk of falling [10]. Serum vitamin D determinants in the Greek population are: age in obese people, season of blood sampling, sex, participation in organized physical activity, and sun exposure in summer months [11].

Quantitative bone ultrasound (QUS) is re-emerging as a low-cost, ionizing radiation-free, simple, and portable screening technique that is able to identify women at risk for osteoporosis and that may be used by general practitioners in primary care. The technique measures the broadband ultrasound attenuation (BUA), expressed in dB/MHz, and the speed of sound (SOS), expressed in m/s. The third variable, the stiffness index (SI), is automatically calculated by the device from the BUA and the SOS. Lower values of the QUS parameters were associated with a significant increase of any subsequent fracture at any site [12]. There are several lifestyle, nutritional, and biochemical factors that affect QUS parameters. Previous studies in healthy Greek women have shown that weight status, physical activity, and sedentary lifestyle may affect BUA, SOS, and SI [13]. Nutritional calcium, vitamin D supplementation, and protein are also correlated with QUS parameters [14,15]. Although there are data on the positive effect of adherence to specific healthy dietary patterns on BMD and bone resorption [16,17,18], there is a literature gap in the effect of dietary patterns on QUS parameters.

The purpose of this study is firstly to identify the dietary patterns that indicate the nutritional habits of Greek adults and secondly, to examine the possible associations of these patterns with serum 25(OH)D levels and QUS phenotypes. The current study is part of the OSTEOS project, an observational cross-sectional study that aims to identify the prevalence of vitamin D deficiency of adults in Greece [11].

## 2. Materials and Methods 

### 2.1. Study Design and Population

The population of the current study was recruited during the OSTEOS study from several rural and urban areas of Greece. The design of this observational cross-sectional study, the collection of demographic data, and the inclusion criteria are detailed elsewhere [11]. Out of the initial population of 970 subjects, the dietary analysis was carried out for 741 subjects due to missing nutritional data in the rest of the population. The subjects did not receive medication for osteoporosis or vitamin D and calcium supplements.

### 2.2. Anthropometric and Biochemical Measurements

The anthropometric characteristics, body weight, height, and body mass index (BMI), were measured with appropriate methods as detailed elsewhere [11]. BMI was classified according to the World Health Organization [19] into four categories: underweight (<18.5 kg/m^2^), normal weight (18.5–24.9 kg/m^2^), overweight (25–29.9 kg/m^2^), and obesity (≥30 kg/m^2^). Heel bone properties were measured using the Achilles quantitative ultrasound (QUS) device as detailed elsewhere [11]. For normative data, we used reference data for the QUS measurements of the calcaneus specific for Greek population [20]. The lower the QUS parameters values, the higher the fracture risk.

Following a 12-hour fast, all subjects had a sample of venous blood withdrawn for serum isolation between 08:00 and 09:00 h. Total calcium (Ca), phosphorus (P), parathyroid hormone (PTH), and 25(OH)D were measured. Intact parathyroid hormone (iPTH) measurements were performed on a Roche/Modular Analytics analyzer, which employs electrochemiluminescence immunoassay technology (ECLIA). Serum 25(OH)D levels were determined by enzyme immunoassay (Immunodiagnostic Systems, 25(OH)D; Boldon, UK). The biochemical analysis methods are analyzed in detail elsewhere [11]. Regarding vitamin D status evaluation, the serum concentration of 25(OH)D is the best marker of an individual’s vitamin D status because it is the major circulating form and reflects the combination of dietary intake and cutaneous skin synthesis. Concerning serum vitamin D thresholds, the following definitions were used: severe deficiency: <25 nmol/L (<10 ng/mL), deficiency: 25–50 nmol/L (10–19.9 ng/mL), insufficiency: 50–75 nmol/L (20–29.9 ng/mL), and sufficiency: ≥75 nmol/L (≥30 ng/mL) [11]. 

### 2.3. Lifestyle Information Assessment

The assessment of hours spent on sedentary activities such as watching television (TV) or working on a personal computer (PC), and engaging in moderate or vigorous organized physical activity were obtained from The International Physical Activity Questionnaire (IPAQ, short version) that was completed under the supervision of the investigator [21]. The hours of sun exposure of the subjects were also evaluated. According to the blood collection day, the samples were divided in two seasons: winter–spring (December until May) and summer–autumn (June until November) as described in the OSTEOS study [11].

### 2.4. Dietary Assessment

Dietary information was collected via a validated, semi-quantitative, 76-item FFQ [22]. All participants reported their daily, weekly, or monthly average intake of several foods during the previous year. Then, the frequency of consumption was quantified on the basis of servings per week, according to the dietary guidelines for adults in Greece [23]. Mixed foods were taken into account and assigned into the respective food groups.

### 2.5. Statistical Analysis

Statistical analysis was conducted using the statistical software package IBM SPSS Statistics for Windows, Version 19.0. Continuous variables were presented as mean ± standard deviation, while categorical variables were presented as relative frequencies. Analysis of variance (ANOVA) was used to examine differences among the groups for different continuous, while the chi-squared test was used to evaluate associations between categorical variables. Independent relationships between serum vitamin D levels, or QUS parameters and other variables as well as the interactions were assessed by stepwise linear regression and univariate general linear model (GLM). All tests were two-sided with a significance level < 0.05.

Concerning the dietary analysis, the transformations of foods and food groups from daily, weekly, or monthly average intake to servings per week were calculated programmatically in the R programming language. Outliers were defined as values that exceeded 5 SD above or below the mean and were removed from subsequent analyses [24].

Principal component analysis (PCA) was conducted to identify underlying dietary patterns [25]. In order for PCA to be effective in assessing food patterns, strong correlations between the predictive variables should exist. The correlation matrix of the food variables used in the present analysis showed that there were several correlation coefficients (r) > │0.4│, indicating that the variables were highly correlated. Moreover, the Kaiser–Meyer–Olkin test of sphericity result and Barlett’s criterion value (0.76) implied strong interrelationships between food variables and the suitability of the data set for PCA. The orthogonal rotation (varimax option) was used to derive optimal non-correlated components (dietary patterns). From the entire database, 20 food groups were used. To decide the number of components to retain, the Kaiser criterion was used, according to which the number of components to be retained from PCA was equal to the number of eigenvalues > 1. In our analysis, six food patterns were selected. Based on the fact that factor loadings/correlation coefficients represent the correlation of each predicting variable with the dietary pattern score, higher absolute values indicate that the variable contributes more to the construction of this particular pattern. The dietary patterns were named according to the scores of the predicting variables that correlated most with the component/pattern (>│0.4│). The PCA was performed using the statistical software package IBM SPSS Statistics for Windows, Version 19.0. Outliers were defined as values that exceeded 3 SD above or below the mean and were removed from subsequent analyses [26].

A voluntary participation agreement was obtained from each participant. The study was approved by the Ethics Committee of Harokopio University. The Committee also approved the informative and Consent form, attached to the proceedings of session 15/8–12–2005. The paper follows the rules of the Declaration of Helsinki.

## 3. Results

The descriptive characteristics of population are detailed in Table 1. The mean age of our population was 49.8 ± 13.4 years (range 18–86 years) and 89.3% were women. Concerning the nutritional habits of population, six dietary patterns were derived from PCA analysis. From the initial 50 foods and food groups, 20 were included in the PCA because of their high intercorrelation level. Six different diet components explained 52.2% of total variability of the population nutrition. Higher absolute values of the score coefficients derived from PCA indicate that the food contributes more to the development of the component. The components were defined as follows—component 1: the ‘vegetables–fruit’ pattern, which includes cooked and raw vegetables, refined rice, fresh fruits and fish; component 2: the ‘fast-food’ pattern, that consists of refined breads, processed meat, and full-fat cheese; component 3: the ‘Western’ pattern, that is characterized by red meat, refined pasta, potatoes, and poultry; component 4: the ‘healthy’ pattern that includes low-fat milk and yogurt, refined breakfast cereals, non-refined breads, and low-fat cheese; component 5: the ‘sweets’ pattern, with milky and starchy sweets; and component 6: the ‘traditional’ pattern, which includes full-fat dairy products as main component as well as legumes (Table 2). As shown in Table 2, legumes also contribute to pattern 1, as the score coefficient is 0.419 (>│0.4│). 

The correlation among the score of compliance at dietary patterns and other phenotypes is presented in Table 3.

Serum vitamin D levels associated positively with the ‘healthy’ pattern and negatively with the ‘sweets’ pattern (Table 3). After adjustment for age, sex, and BMI, participation in physical activity and hours of summer sun exposure the statistical significance remains for both dietary patterns (B = 0.667, *p* = 0.024 and B = −0.919, *p* = 0.002, respectively).

BMI was correlated positively with adherence to the ‘vegetables–fruit’ and ‘Western’ pattern (Table 3). After adjustment for sex and age, hours of sedentary activities (TV watching, PC activity), and hours of participation in organized physical activities, BMI was correlated positively only with adherence to the ‘Western’ pattern (B = 0.532, *p* = 0.010).

After adjustment for age, BMI and sex, the positive effect of the ‘fast-food’ dietary pattern on BUA was not significant (B= −0.2, *p* = 0.754) and the positive effect of the ‘healthy’ pattern remained (B = 1.619, *p* = 0.008).

Subjects participating more in organized physical activity had better compliance to the ‘healthy’ pattern regardless of age, sex, and BMI (B = 3.704, *p* = 0.000).

**Figure 1:**
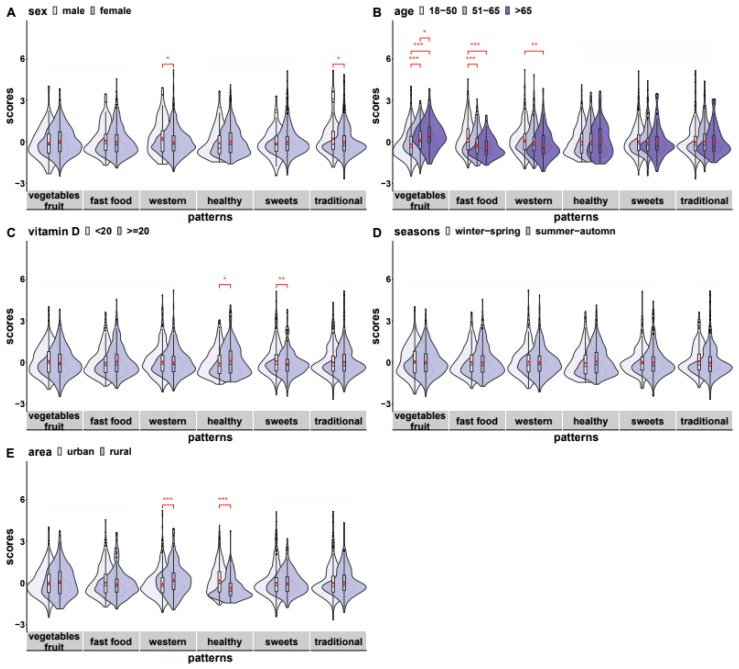
Distribution of scores derived from PCA (principal component analysis) among; (**A**) sex, (**B**) age groups, (**C**) serum vitamin D status, (**D**) season of blood sampling, and (**E**) area of residence. * *p* < 0.05, ** *p* < 0.01, *** *p* < 0.001, • mean, **₋** median.

The distribution of scores derived from PCA and the compliance in specific patterns according to sex, age group, vitamin D status, and area of residence are presented in Figure 1.

Males were more engaged in the ‘Western’ pattern than females. This difference remained after adjustment for age (B = 0.255, *p* = 0.032) as well. Moreover, males had better compliance to the ‘traditional’ pattern after adjustment for age (B = 0.258, *p* = 0.034).

Urban living subjects had lower compliance to the ‘Western’ pattern than rural living subjects and after adjustment for sex, age, and BMI the difference remained significant (B = −0.266, *p* = 0.001).

Urban residents achieved higher scores in the ‘healthy’ pattern than those living in rural areas, and the difference remained after adjustment for age, sex, and BMI (B = 0.562, *p* = 0.001).

Variables that correlated in simple statistical tests were entered to Univariate GLM with a stepwise procedure to evaluate serum 25(OH)D and bone QUS phenotypes’ determinants. The models presented in Table 4, Table 5, Table 6 and Table 7 resulted from the above data. Concerning the serum 25(OH)D determinants, when all correlates were included in the model, the season of blood sampling and the adherence to the ‘healthy’ pattern lost their significance. However, when investigating how two determinants interact, it was derived that the adherence to a healthy dietary pattern in winter months had a positive effect on vitamin D levels. In summer months the adherence to the ‘healthy’ dietary pattern did not influence vitamin D levels (Table 4).

Concerning BUA determinants, when the ‘healthy’ dietary pattern was included in the regression, the participation in organized physical activity lost statistical significance as determinant of BUA, due to its correlation with healthy dietary pattern. As a result, only the ‘healthy’ pattern was included in the model.

## 4. Discussion

This study investigates how nutrition is associated with serum vitamin D levels as well as the bone health parameters BUA, SOS, and SI, taking into consideration lifestyle parameters. Although BMD is the main predictive risk factor for an osteoporotic fracture, measurement of quantitative ultrasound (QUS) has been found to be associated with bone fragility and increased fracture risk [27]. The ultrasound attenuation (BUA) is due to its absorption and dispersion into the bones and soft tissues. It is affected by both bone density and structural parameters. Wave velocity (SOS) is affected by bone density and tissue elasticity. Therefore, the method evaluates bone density (BMD) and bone structure. These measurements are correlated with bone density (as measured by the dual-energy X-ray absorptiometry: DXA method) and can be used to categorize individuals in osteoporotic and healthy, while it seems that the QUS technique can predict fracture risk, especially in older women. The lower the QUS parameters values, the higher the fracture risk [28,29]. The method was chosen because it provides information on density as well as on the quality and architecture of the bone tissue. In addition, the method is economical and fast, while the measuring device is portable and the subject is not exposed to ionizing radiation [29].

Six dietary patterns explain 52.2% of variability of Greek adults’ nutritional habits. A prudent dietary pattern like the ‘vegetables–fruit’ pattern explains the biggest rate of variability, a positive fact for the current population. A dietary pattern with similar composition was the most dominant food pattern for another Greek adult population [30] as well. Even though in other studies [31,32,33] a positive association was described between BMD and a dietary pattern was characterized by high consumption of fruit and vegetables, in the current study the adherence to a pattern rich in vegetables, fruits, rice and fish did not reach the statistical significance and it did not correlate with heel QUS parameters. A study on older adult population suggested that better compliance to a ‘prudent’ diet score with fruits, vegetables, and oil fish at early old age predicts bone size on average 11 years later [34].

The ‘Western’ pattern, as expected, related with higher BMI values and men are more susceptible to this pattern than women. In addition, males consume more full-fat dairy products than females, the main component of the ‘traditional’ pattern.

Urban residents have better compliance to the ‘healthy’ and less to the ‘Western’ pattern than those living in rural areas. Other studies that have compared the dietary patterns of rural versus urban adults also indicated that rural adults had poorer dietary habits than urban adults [35]. Rural adults participate in less moderate-to-vigorous physical activity and have poorer dietary behaviors than their urban counterparts, a fact that may contribute to their higher rates of obesity [36]. Residency in a rural area usually implies healthier eating and physical activity habits; nevertheless, sometimes rural residents may not have the expected lifestyle [37].

The more hours the subjects participate in organized physical activity, the higher the score they achieve in the ‘healthy’ pattern. Moreover, participation in organized physical activity parameter loses statistical significance as determinant of BUA due to its correlation with the ‘healthy’ pattern. These two parameters together may reflect a healthier lifestyle that could be further examined by evaluation of lifestyle patterns. This beneficial effect of the ‘healthy’ dietary pattern on BUA may be due to low-fat dairy products and breakfast cereals, included in the pattern. Dairy products contain or are fortified with significant proportions of nutrients important for bone health (calcium, protein, vitamin D, etc.) [38]. Breakfast cereals are also usually enriched with vitamins and minerals and they are a plant source of protein associated with bone health [39]. SOS and SI parameters are not affected by dietary patterns.

In a recently published review of 49 studies it was concluded that a dietary pattern that emphasized the intake of fruit, vegetables, whole grains, poultry and fish, nuts and legumes, and low-fat dairy products and de-emphasized the intake of soft drinks, fried foods, meat and processed products, sweets and desserts, and refined grains was beneficial for bone health [40].

Positive determinants of serum vitamin D levels are adulthood, hours of summer sun exposure, and participation in organized physical activity. Adherence to the ‘healthy’ pattern, which protects vitamin D levels only in winter months when serum levels decline, may be due to the enrichment of dairy products and breakfast cereals with vitamin D. BMI and the ‘sweets’ pattern are negative determinants. This study confirms the results of other studies which investigated vitamin D determinants [41,42,43].

Positive BUA determinants are male gender, BMI, adulthood, and adherence to a ‘healthy’ pattern. SOS negative determinant is age. Vitamin D levels greater than 20 ng/mL have a protective effect on SOS parameters from sedentary activity. Recently published data from the OSTEOS study associate QUS parameters with vitamin D status; specifically, individuals with 25(OH)D ≥ 20 ng/mL had higher SOS than those with 25(OH)D < 20 ng/mL [11]. SI positive determinants are organized physical activity and adulthood.

A Greek study of all age group women demonstrates that adulthood, BMI, and organized physical activity are positive determinants of QUS parameters, the results of which are similar to the current study [13].

Many studies identify associations between individual nutrients or food items and musculoskeletal health outcomes [44]. Use of individual nutrients does not consider interactions and cumulative effects between different nutrients and foods. Dietary pattern analysis has been used to overcome these limitations by studying the overall diet rather than the intakes of individual nutrients. This is particularly important for disease prevention or treatment because the small effect of a single or a few nutrients may not be able to be detected, while dietary pattern analysis considers the joint effects of nutrients and foods based on overall eating behavior. The results of dietary patterns studies can also be easily translated into distinct dietary guidelines for the public.

This current study has several limitations: it is an observational, cross-sectional study and it is not appropriate to draw causal effect implications or to generalize the results from this population. Residual confounding may also exist because of unmeasured variables. Another limitation of the study is the self-reported medical history and medication data. The consumption of vitamin D-enriched food was not determined by the current study. One last limitation is the method used for the determination of serum 25(OH)D levels: enzyme immunoassay, a globally used method. The gold standard is mass spectrometry, which is not accessible everywhere. Most other methods have a 100% cross reactivity between 25(OH)D and 24,25-dihydroxyvitamin D. On the other hand, the study has also several strengths: it used a large national representative population of urban and rural citizens, with a vast age range.

## 5. Conclusions

A prudent dietary pattern consisting of vegetables, fruits, rice, and fish, explains more of the nutrition variability of the current population. Several determinants explain part of the variability of 25(OH)D, BUA, SOS, and SI. The modifiable factors that can positively affect QUS parameters and vitamin D levels are participation in organized physical activity, reduced hours of sedentary activities, achievement of healthy BMI, healthy dietary habits, and reduced sweet consumption. More studies are needed, with larger samples and a greater number of biochemical, lifestyle, and genetic parameters to determine individualized guidelines and promote public health and prevention. 

## Figures and Tables

**Table 1:** Descriptive characteristics. PTH: parathyroid hormone; BMI: body mass index; BUA: broadband ultrasound attenuation; SOS: speed of sound; SI: stiffness index.

	Total (Mean ± SD)	Males (Mean ± SD)	Females (Mean ± SD)	*p*-Value
*n*	741	79	662	
Age (years)	49.8 ± 13.36	45.26 ± 16.07	50.40 ± 12.85	0.003
25(OH)D (ng/mL)	20.01 ± 7.98	22.66 ± 8.95	19.63 ± 7.76	0.002
PTH (pg/mL)	40.19 ± 15.57	39.67	40.16 ± 15.42	0.772
ΒΜΙ (kg/m^2^)	27.58 ± 5.53	27.98 ± 5.21	27.52 ± 5.57	0.451
BUA dB/MHz	114.53 ± 16.32	118.73 ± 19.35	113.81 ± 15.65	0.023
SOS m/s	1552.49 ± 87.92	1555.29 ± 159.71	1551.98 ± 70.61	0.849
SI	90.94 ± 19.62	97.5 ± 25.7	89.96 ± 18.32	0.019
Sun exposure summer (hours/day)	12.48 ± 10.93	17.87 ± 13.86	11.81 ± 10.33	0.00
TV watching or PC activity (hours/day)	3.02 ± 2.14	3.19 ± 2.39	3 ± 2.11	0.404
Organized physical activity moderate and/or vigorous (minutes/day)	12.55 ± 27.68	16.51 ± 30.70	12.03 ± 27.29	0.137

**Table 2:** Dietary patterns derived from principal component analysis (PCA).

	Components					
Food Group	1. Vegetables–Fruit	2. FAST FOOD	3. Western	4. Healthy	5. Sweets	6. Traditional
Non refined breads	0.204	−0.123	−0.118	**0.529**	0.131	−0.118
Refined breads	0.016	**0.848**	0.119	−0.068	0.101	0.023
Refined breakfast cereals	−0.076	−0.001	−0.022	**0.706**	0.058	0.349
Full-fat cheese	0.197	**0.659**	0.106	−0.019	0.169	0.052
Low-fat cheese	0.099	0.189	−0.036	**0.485**	−0.283	−0.191
Fish	**0.494** *	−0.162	0.154	0.032	−0.109	−0.083
Fresh fruits	**0.521**	0.013	−0.158	0.230	0.333	0.046
Legumes	0.419	0.081	−0.029	−0.014	−0.152	**0.447**
Poultry	-0.028	0.111	**0.555**	0.364	−0.189	-0.143
Red meat	0.118	0.146	**0.767**	−0.032	−0.071	0.033
Full fat milk and yogurt	−0.049	−0.007	0.107	−0.035	0.044	**0.829**
Low-fat milk and yogurt	0.077	−0.002	−0.042	**0.729**	−0.008	−0.039
Refined pasta	0.116	0.088	**0.629**	−0.140	0.158	0.148
Refined rice	**0.558**	0.072	0.299	0.072	−0.019	0.179
Milky sweets	−0.026	0.064	0.149	0.061	**0.784**	−0.083
Starchy sweets	−0.114	0.284	0.000	−0.068	**0.534**	0.055
Cooked vegetables	**0.751**	0.023	0.075	0.004	−0.039	0.083
Raw vegetables	**0.669**	0.085	0.005	0.73	−0.037	−0.125
Processed meat	−0.141	**0.818**	0.120	0.110	0.010	−0.043
Potatoes	0.069	0.043	**0.612**	−0.201	0.212	0.018

* Score coefficient in bold indicates the food group contributes to pattern.

**Table 3:** Correlation between dietary patterns and continuous variables.

*n* = 741	1. Vegetables–Fruit	2. Fast Food	3. Western	4. Healthy	5. Sweets	6. Traditional
Continuous Variables	**r (*p*-value)**	**r (*p*-value)**	**r (*p*-value)**	**r (*p*-value)**	**r (*p*-value)**	**r (*p*-value)**
Age (years)	**0.244** * (0.00)	**−0.272** (0.00)	−0.116 (0.002)	0.017 (0.65)	−0.061 (0.099)	−0.029 (0.430)
25(OH)D (ng/mL)	−0.066 (0.074)	0.017 (0.653)	−0.05 (0.173)	**0.107** (0.004)	**−0.119** (0.001)	0.018 (0.628)
PTH (pg/mL)	**0.101** (0.007)	**−0.077** (0.039)	−0.011 (0.773)	**−0.141** (0.00)	**0.086** (0.022)	−0.001 (0.974)
ΒΜΙ (kg/m^2^)	**0.074** (0.046)	−0.025 (0.498)	**0.094** (0.012)	−0.055 (0.144)	−0.058 (0.120)	0.004 (0.922)
BUA dB/MHz	−0.035 (0.394)	**0.102** (0.014)	0.067 (0.105)	**0.089** (0.03)	0.047 (0.256)	0.001 (0.980)
SOS m/s	−0.054 (0.199)	0.012 (0.778)	−0.009 (0.823)	0.046 (0.277)	0.075 (0.074)	−0.001 (0.974)
SI	−0.079 (0.084)	0.071 (0.121)	**0.098** (0.032)	0.046 (0.320)	−0.006 (0.893)	0.016 (0.721)
Sun exposure summer (hours/day)	0.040 (0.287)	0.068 (0.070)	0.011 (0.778)	0.05 (0.182)	0.005 (0.904)	0.050 (0.182)
TV watching or PC activity (hours/day)	−0.027 (0.481)	−0.036 (0.349)	−0.022 (0.562)	0.032(0.396)	0.033 (0.383)	−0.04(0.293)
Organized physical activity moderate and/or vigorous (minutes/day)	−0.011 (0.763)	0.027 (0.459)	0.024 (0.526)	**0.146** (0.00)	−0.021 (0.564)	0.037 (0.318)

* Coefficients in bold indicate statistical significance.

**Table 4:** Determinants of serum 25(OH)D.

Variable	B (Standard Errors)
Intercept	20.993 (1.804) ***
BMI (kg/m^2^)	−0.157 (0.054) **
Age group (reference > 65 years)	
18–50 years	1.948 (0.959) *
61–65 years	1.847 (0.986)
Summer sun exposure (hours/day)	0.091(0.027) ***
Organized physical activity moderate and/or vigorous (minutes/day)	0.042 (0.011) ***
‘Healthy’ dietary pattern adherence * Winter	1.058 (0.497) *
‘Healthy diet’ dietary pattern adherence * Summer	0.441 (0.358)
‘Sweets’ dietary pattern adherence	−0.911 (0.293) **
Adjusted R-squared	0.081

* *p* < 0.05, ** *p* < 0.01, *** *p* < 0.001.

**Table 5:** Determinants of BUA (broadband ultrasound attenuation).

Variable	B (Standard Errors)
Intercept	83.906 (3.816) ***
Sex (reference female)	
Male	5.189 (1.877) **
BMI (kg/m^2^)	0.571 (0.112) ***
Age group (reference >65 years)	
18–50 years	19.310 (2.204) ***
61–65 years	8.662 (2.289) ***
Adherence to the ‘healthy’ food pattern	1.878 (0.615) **
Adjusted R-squared	0.197

* *p* < 0.05, ** *p* < 0.01, *** *p* < 0.001.

**Table 6:** Determinants of SOS (speed of sound).

Variable	B (Standard Errors)
Intercept	1614.329 (14.118) ***
Age (years)	−1.086 (0.271) ***
TV watching or PC activity (hours/day) * 25(OH)D < 20 ng/mL	−5.348 (1.821) **
TV watching or PC activity (hours/day) * 25(OH)D ≥ 20 ng/mL	−0.960(2.130)
Adjusted R-squared	0.041

* *p* < 0.05, ** *p* < 0.01, *** *p* < 0.001.

**Table 7:** Determinants of SI (stiffness index).

Variable	B (Standard Errors)
Intercept	75.188 (2.416) ***
Organized physical activity moderate and/or vigorous (minutes/day)	0.065 (0.029) *
Age group (reference > 65 years)	
18–50 years	22.402 (2.638) ***
61–65 years	8.352 (2.728) **
Adjusted R-squared	0.190

* *p* < 0.05, ** *p* < 0.01, *** *p* < 0.001.
